# A qualitative study to identify thematic areas for HIV related patient-reported outcome measures (PROM) and patient-reported experience measures (PREM)

**DOI:** 10.1186/s41687-023-00582-y

**Published:** 2023-05-01

**Authors:** Anna-Leena Lohiniva, Sanna Isosomppi, Sini Pasanen, Jussi Sutinen

**Affiliations:** 1 Finnish Institute for Health and Welfare, Helsinki, Finland; 2 HivFinland (patient organization), Helsinki, Finland; 3 Department of Infectious Diseases, Inflammation Center, Helsinki University Hospital and University of Helsinki, Helsinki, Finland

**Keywords:** HIV, Patient-centered care, PROM, PREM

## Abstract

**Background:**

The use of patient-reported outcome measures (PROM) and patient-reported experience measures (PREM) provide health providers with valuable feedback on how to improve clinical care and patient outcomes. This paper describes a qualitative study that was conducted to learn about factors influencing the well-being of people living with HIV (PLHIV) in Finland. The findings will be used to develop themes for HIV-specific PROM and PREM questions.

**Methods:**

PROMs and PREMs were developed by the Finnish Institute for Health (THL) as a part of a project to develop a national quality-of-care registry for HIV. The study aimed to identify issues and concerns among people living with HIV (PLHIV) that influence their well-being (PROMs) and their experiences in the healthcare system (PREMs). The data were collected through face-to-face in-depth interviews and focus group discussions based on open-ended and semi-structured questions. The data were analyzed using thematic analysis.

**Results:**

The assessment identified the following PROMs of concern: psychological well-being, concerns about stigma, physical health, social well-being, sexual well-being, medication uptake, managing other medications with antiretrovirals (ARVs), and growing old. The assessment identified the following PREMs: helping patients understand their own health status, proving an opportunity for patients to discuss physical health, psychological and sexual well-being, supporting the uptake of ARVs, assisting patients with medication use, showing compassion towards patients, and empowering patients against stigma.

**Conclusion:**

These findings of the study can be used to develop domain-specific PROM and PREM questions for the national HIV quality care register.

**Supplementary Information:**

The online version contains supplementary material available at 10.1186/s41687-023-00582-y.

## Background

People living with HIV have various symptoms and concerns despite advances in antiretroviral therapy (ART) [1]. Their well-being is linked to a number of interconnected dimensions including physical, psychological, social, spiritual, and socioeconomic factors [2]. Although global HIV initiatives are still heavily focused on diagnosis, treatment, adherence, and viral suppression, new patient-centered care initiatives are increasing worldwide that look beyond these issues to promote well-being and a more personalized outcome approach [3]. In practice, patient-centered care means that patients can access the care they need and that health providers deliver high-quality care that is responsive to the needs of patients [4, 5].

Patient-reported outcome measures (PROMs) are increasingly used to promote patient-centered care and are important means to monitor the quality of care, while patient-reported experience measures (PREM) are critical for patient-centered care by providing feedback to healthcare providers about the health status of patients and health care services based on patients’ interpretation [6–8]. PROMs provide direct information about the status and health conditions of patients without the interpretation of clinicians, thereby capturing patients’ understanding of their illness and health. PREMs are patients’ direct interpretations of the quality of care they receive. The use of PROMs and PREMs has been shown to improve patient care by detecting issues and addressing patients’ care priorities as well as improving patient-clinician communication and patient engagement [9, 10].

PROMs and PREMs are of particular importance for those with long-term conditions, such as people living with HIV (PLHIV) for whom longitudinal monitoring of their health and well-being is important. HIV-specific PROMs have been developed that are more closely associated with HIV than generic PROMs, which makes them better suited for detecting minor changes in HIV-specific matters, such as HIV-related stigma, comorbidities, and ART-related treatment [11]. Currently, there are no agreed-upon standards for measuring HIV PROMs and PREMs. Instead, there are a number of HIV PROMs and HIV PREMs that have been designed to measure a variety of perceptions and experiences, including health-related quality of life (HRQoL), adherence-related experiences, health care service experience (PREMs), mental health challenges, physical symptoms, coping strategies, HIV self-management, and self-care capability, body image, social support, and issues related to sexual and reproductive health and disability [12–14]. All of them have some shortcomings such as a lack of validation of psychometric properties [14].

PROMs and PREMs are context-specific as they are influenced by a number of cultural, social, and environmental factors such as epidemiological situations, access to care and treatment, and the level of stigma and community support. For example, in a high-prevalence setting, the community may be more accustomed to HIV, and accordingly, PLHIV may be better supported by the surrounding communities than people in low-prevalence settings. In some communities, cultural and religious beliefs may lead to the stigmatization of PLHIV whereas in other settings PLHIV may lack access to care and treatment [12]. Therefore, it is essential to conduct studies to define, validate, and identify which PROMs should be used by professionals caring for PLHIV.

Not much is known about the factors influencing the well-being of PLHIV living in Finland, which is a low HIV prevalence setting. In 2021 there were 161 new infections (2.9/100,000), which is approximately 30 cases more than the previous year. The majority of the cases (59%) were in the Helsinki Metropolitan area. Although new infections were detected across all age groups, the majority were among those 30–34 years and 35–39 years. As much as 6% of the new HIV infections were among those over 65 years old. The majority of new infections were among males (73%). Of the new cases, 73% were among foreign populations residing in Finland and 49% had known about the infection before coming to Finland. The majority of the new HIV infections were detected via testing in public healthcare facilities (83%). HIV testing is also carried out in private-sector healthcare facilities and in voluntary counseling and testing facilities. In half of the new cases (50%) the modes of transmission were not known. In the other half, the reported mode of transmission was heterosexual transmission (21%), men having sex with men (23%), injecting drug use (6%), and mother-to-child transmission (1%). In recent years, localized HIV epidemics have occurred among injection drug users [15]. Free care and treatment are provided by the public sector infectious disease clinics which usually includes one yearly visit to the clinics; additional visits and telephone consultations can be scheduled based on need. As doctors and nurses have only limited opportunities to interact with PLHIV, PROM and PREM questions provide valuable information about the well-being of PLHIV which in turn allows doctors and nurses to focus on issues of importance to patients during the short clinic visits. Providing services of equal quality in low prevalence settings can be challenging as some clinics serve only a few PLHIV so their service and care routines may not be as established as clinics that more regularly serve PLHIV. PROM and PREM allows policymakers to monitor and compare the well-being of PLHIV nationally and adjust their services accordingly.

The Finnish Institute for Health (THL) initiated the development of PROMs and PREMs for PLHIV as a part of their pilot project to develop a national quality of care register for HIV. Developing the PROM and PREM questions was a multi-phased process including the identification of the thematic areas for well-being through a qualitative study and a scoping review, followed by question development based on multi-stage consensus building by a group of multidisciplinary experts including a cultural anthropologist, infectious disease doctors, public health experts, and representatives of patient organizations. Knowledge co-creation was used by engaging PLHIV via in-depth interviews and focus group discussions to validate the acceptability and clarity of the final set of questions [16] followed by a validation exercise to measure psychometric properties.

This paper describes the first step of the PROM development process: the qualitative assessment. The qualitative assessment aimed to identify and understand the primary concerns and issues among PLHIV that influence their well-being in Finland which served as the thematic areas for the development of HIV PROMs and PREMs.

## Methodology

This study utilized qualitative research methodology as the aim of the study was to gain an in-depth understanding of factors that influence the well-being of PLHIV (PROM) and factors that are deemed important for good quality of care (PREM). The study was based on open-ended and semi-structured, qualitative in-depth interviews (IDIs) and focus group discussions (FGDs). IDIs explored personal experiences whereas FGDs, which are commonly used to explore social norms in particular, focused on prevailing perceptions among PLHIV. IDI and FGD guides included probes based on factors influencing the quality of care of PLHIV identified in the recent quality of care-related studies among PLHIV in Finland. Accordingly, the question guides included the following thematic areas: physical health, psychological health, sexual well-being, social well-being, stigma, and side effects of antiretrovirals (ARVs) [17–21]. The interviews included open-ended questions related to issues influencing the well-being of the respondents, which allowed new emerging issues to be included in the study. Each topic was then discussed in detail by asking respondents to describe situations where they confronted that particular issue and their overall impressions about it. The IDIs and FGDs also focused on extensive probing such as “Tell me more,” “Please explain what you mean,” to generate open discussions and to gain a more in-depth understanding of the issues. The question guides for IDI and FGD can be found in the supplementary material.

Sampling was based on maximum variation to gain diversity among PLHIV in terms of age, gender, nationality, geographic location in the country, mode of transmission of HIV, and time since HIV diagnosis [22]. The recruitment of study participants was based on snowballing in different geographic locations in Finland (South, North, West, and East) through patient organizations and various secondary and tertiary infectious diseases clinics that were asked to recruit diverse PLHIV to capture gender, different ages, and ethnic backgrounds. Each organization and the clinics were asked about PLHIV with certain characteristics to ensure that the study sample include various types of participants. Data saturation was used to obtain an adequate sample size. The minimum sample size for initial data collection was 50 IDIs followed by three additional interviews until new themes were no longer produced [23]. The initial sample size was based on a literature review that indicated large qualitative studies often comprised 50 to 60 people [24]. Adults and youth (+ 15 years old) living with HIV who were currently under the care of an infectious disease specialist were eligible to join the study.

A qualitative researcher (A-LL) conducted the IDIs, whereas FGDs were conducted jointly by the qualitative researcher and infectious disease doctor (JS) who acted as a note taker and moderator, respectively. All IDI and FGDs were conducted between October 2019 and February 2020. Data collection began by piloting and modifying question guides based on debriefing sessions conducted by the data collection team followed by an average of 1–2 IDIs or FGDs per day. All interviews were conducted in Finnish or English. A translator was used for Russian and Thai respondents. The interviews started with friendly opening questions to establish rapport between the respondents and researchers, followed by questions from the guide with extensive probing and an open-ended closing question. Each IDI lasted approximately 40 min while each FGD took from 60 to 90 min. All IDIs and FGDs were conducted in a private space in the infectious disease clinics or on the premises of patient organizations. A few interviews were conducted by phone based on the preference of the study participants. No identifiers or recordings were collected to ensure privacy and confidentiality. The question guides can be found in Table 1.

**Table 1:** Characteristics of the study participants

Variable	N = 65	%
**Gender**		
Women	34	52%
Men	31	48%
**Nationality**		
Finnish	36	55%
Other nationalities	29	45%
**Age**		
Between 18 and 49 years old	33	50%
Between 50 and 64 years old	29	45%
65 years and older	3	5%
**HIV diagnosis**		
more than 10 years ago	39	60%
Less than 10 years ago	26	40%
**Geographic location**		
Helsinki Capital area	52	80%
Other locations	13	20%

The study followed thematic analysis with purposeful data reduction activities [25, 26]. The analysis was based on IDI and FGD notes taken by the qualitative researcher experienced in note taking. The researcher took short notes during the interviews and expanded them to long notes the same day to avoid recall bias. The long notes also included direct quotes and contextual descriptions of each interview to increase the depth of the data [27]. The rigor and validity of the notes were established by reviewing the short notes with the study participants at the end of each IDI and FGD to ensure that the key points and meanings were understood. In addition, the interviewer and notetaker discussed the notes and their first impressions during a debriefing session after each FGD. The analysis was carried out using inductive thematic analysis that also considered emerging themes [28]. The process started with data familiarization during which the analyst read the data multiple times to get an overall idea of the dataset and to create an initial set of codes that resulted in a codebook. Coding was conducted for each interview using the codebooks, emerging new codes were also included using NVIVO12, followed by refining and expanding codes and developing categories. The initial analysis was shared with the study team including the notetaker to get a consensus on the emerging categories and the ways to explore relationships and patterns across the interviews. Discussions were carried out among team members until a consensus was reached. In the final stage, the analyst developed the interpretation. The syntheses of the results served as the foundation for thematic areas for PROMs and PREMs [29]. The consolidated criteria for reporting qualitative research checklists were used to ensure the quality of reporting the results [30].

The research was exempted from an ethical review by the Finnish Institute for Health and Welfare as the data is anonymous. All interviews and FGDs started with verbal consent. All interviews were conducted in a private space. No compensation was provided.

## Results

### Characteristics of study participants

The study participants included 77 PLHIVs of various ages and nationalities from a variety of locations across Finland. The sample included individuals who have known about their HIV diagnosis for more than 10 years and those who learned about their HIV diagnosis more recently. The participants had contracted HIV through sex, by injecting drugs, and from mother-to-child transmission. The sample included four focus group discussions with 8–12 participants and the rest were in-depth interviews. The sample characteristics can be found in Table 1.

### Issues and concerns in life (PROM areas)

Participants were asked what concerns and issues were important for their well-being. They described concerns across eight domains: psychological well-being, concerns about stigma, physical health, social well-being, sexual well-being, medication uptake, managing other medications with ARVs, and growing old. The topics are not presented in any particular order.

### Psychological well-being

Most participants perceived psychological well-being as of utmost importance because it was linked to the other seven issues and concerns in life (PROM areas).

For example, many of them described their experiences with depression or anxiety which influenced their willingness to continue taking ARVs or being in contact with other people.

Participants highlighted that critical to their psychological well-being was knowing the level of their viral load, knowing that their medication (ARV) was working and that their current medication was the best possible fit for them.

***“It is always in the back of my mind. How well I am doing? It is always a relief to know that my medication is working.” (A 50-year-old woman)***.

### Experiences of stigma

Most experiences of stigma were related to healthcare settings outside of infectious disease clinics. Stigma manifested in health care provider attitudes and actions such as overuse of personal protective barriers, scheduling or changing appointments to be the last one of the day, blaming the patient for their HIV status, calling them names, and denying care. A few experiences of stigma related to family and friends include distancing and gossiping.

***“When they knew I had HIV, they refused to give me an appointment for dental care.” (A 46-year-old man)***.

### Fear of stigma

Respondents feared being abandoned or rejected if their HIV status was known to friends, family, or the community at large. They also feared being pitied or the subject of gossip. In addition, respondents feared that the negative attitudes towards them could expand to include other family members including their children. Many respondents had not disclosed their status to anyone.

***“I am worried that people will pity me and maybe my family will be worried. That’s why no one but doctors and nurses know about my HIV status.” (A 47-year-old woman)***.

### Self-stigma

Some respondents explained having negative attitudes towards themselves that manifested in self-hate, fear of dying, and an inability to accept their HIV diagnosis. Respondents further explained that stigma also influenced their willingness to seek care and affected their psychological well-being and social well-being leading to a reluctance to disclose their HIV status and the continuous fear that their status will be discovered.

### Physical health

Most participants explained that physical health was of great importance. However, most considered their health fairly good and their overall physical health problems did not limit their day-to-day routines. They cited physical health problems that were linked to other chronic conditions or to other infectious diseases, and many respondents pointed out those physical problems could create psychological problems and vice versa, which made physical health an important issue.

***“I evaluate my health as relatively good. I do not have any major issues except those that are associated with my age. But I really need to take care that it remains this way.” (A 62-year-old man)***.

### Social well-being

The importance of social well-being was mostly linked to participants’ relationships with their partners, and family members’ acceptance of their HIV diagnosis. Most respondents explained that fear of being stigmatized and previous stigmatizing experiences influenced their willingness to build relationships with people or disclose their status.

***“I haven’t told anyone. Only my doctor and the nurses know. I couldn’t handle the rejection of my family.” (A 55-year-old man)***.

### Sexual well-being

Participants were divided regarding the importance of sexual well-being. Some people believed that it influenced their well-being significantly whereas others believed that it did not. Those who perceived it as significant explained that sexual well-being was linked with their relationship with their partner, transmission fears, or gynecological problems. They also noted that sexual well-being meant being well informed about pregnancy, abortion, and medications to increase sexual potency.

***“For me, sexual well-being also means my overall relationship with my partner. It is a group of things that makes my sexual life satisfactory.” (A 35-year-old man)***.

### Uptake of ARVs

Respondents were also divided in their views regarding the importance of uptake of medications for their overall well-being. Some respondents believed that it can relatively rapidly influence their viral load whereas others believed that forgetting once in a while or even frequently did not have much influence on their health or well-being. Many respondents had experienced times when uptake of medication was difficult.

***“Last year I was tired of taking my medication. I was down all the time. It passed though and I am better now.” (A 30-year-old woman)***.

### Managing other medications with ARVs

Respondents explained that it was essential for their well-being to understand how other medications such as those for chronic conditions, infections, mental health problems or vitamins worked with ARVs. Respondents explained that using medications that are not compatible with ARVs could lessen the effects of ARVs, impact their overall health status, or create new health problems and symptoms.

***“I had these blood pressure medications, and I was not sure if I should stop them or continue them with the ARVs.” (A 52-year-old woman)***.

### Aging with HIV

Some elderly respondents worried about their ability to adhere to ARVs in the future due to memory problems or other disabilities characteristic of elderly people. They were also concerned about the compatibility of ARVs with the growing number of other medications that they expect to use as they age. The respondents also shared concerns about the lack of elderly caretakers trained on the special needs of PLHIV. In addition, respondents worried about stigma and discrimination leading to a denial of admission to elderly homes or having their HIV status disclosed to others without consent.

**“Yes, I am thinking about who will care for me and if they will be able to care for me.” (A 65-year-old man)**.

### Issues and concerns related to HIV care (PREM areas)

Participants were asked what factors make the quality of HIV care optimal. They included six domains: helping patients understand their own health status, providing an opportunity for patients to discuss physical health and psychological and sexual well-being, supporting the uptake of ARVs, assisting patients with medication use, showing care towards patients, and empowering patients against stigma.

### Helping patients understand their own health status

The first factor most participants mentioned as a sign of good care was having health care providers who could explain their health status in a way they could understand. This included what the viral load level means, how their medications are working, and how their medications compare with the latest medical innovations in the world. Participants explained that concern about their HIV status was always present even if ARVs worked well. They noted that there was always hope that a cure for HIV was found, which made the discussions about the latest innovations important.

***“In the back of mind, I always have the hope that cure for HIV will be discovered. If doctors do not share what they know about the new medications I am not satisfied.” (A 55-year-old man)***.

### Showing care toward patients

The majority of the respondents explained that good HIV care gave them a feeling that the health care providers cared about their health and well-being. Respondents explained that the question “How are you doing?” was of utmost importance to them as it gave them a feeling that the health providers truly care about them.

***“When the doctor asks how I am doing, it makes me feel good and cared for. It also gives me the chance to open a discussion about anything.” (A 52-year-old man)***.

### Having an opportunity for patients to discuss physical health, psychological and sexual well-being

Many respondents explained that discussions with infectious disease doctors about various physical health problems were vital to ensure whether or not their health problems are HIV-related. In addition, if they were experiencing any health problems, a referral from an infectious disease doctor to another specialist was much appreciated as they were trusted health providers and were expected to refer patients to the best possible specialists. Some respondents cited having no one else to talk to about their health problems.

***“I like discussing everything with the infectious disease doctor. I trust them. I am sure they provide me with the best possible advice.” (A 38-year-old man)***.

Many respondents also mentioned that talking about psychological problems was a relief and many mentioned that they had no one else to talk to if they were feeling down. Respondents clarified that the expectation was not to have therapeutic care discussions with the doctor, but that it provided them with a unique opportunity to discuss how to improve their psychological well-being or to get referrals to specialists if needed.

***“For me it is important that I can talk about everything when I’m at the clinic. We search for solutions to my problems together with my doctor.” (A 35-year-old man)***.

Some respondents clarified that they appreciated having an opportunity to bring up issues related to sexual well-being with their health care providers, especially when they had no one else to talk to about sexual health-related matters.

***“With a gynecologist, I can talk about medical problems, but I cannot have a discussion about sexual health in general.” (A 35-year-old woman)***.

### Supporting the uptake of ARVs

Most respondents highlighted the importance of having a discussion about their uptake of ARVs during the clinic visit, which helped them take ARVs more seriously. Respondents noted that this was of particular importance during the times when taking ARVs was difficult, which happened from time to time.

***“Doctors cannot help me remember to take medications but it helps me understand that taking ARVs is very important. Sometimes I get tired of taking them.” (A 45-year-old man)***.

Although respondents, in general, did not complain about the side effects of their current ARVs, they frequently confirmed that the infectious disease clinic was the only place where they could discuss the issue and change the medication if needed.

***“This is the only place to talk about ARV. They are experts here.” (A 62-year-old man)***.

### Assisting patients with medication use

Most respondents explained that infectious disease doctors play a vital role in advising them about the use of all kinds of medications to ensure that they were compatible with ARVs. They explained that the infectious disease clinics were the only trusted sources of such information.

***“I always get in touch with the infectious disease clinic if I get a medication. It is very important. I call them.” (A 63-year-old man)***.

### Empowering patients against stigma

Many respondents mentioned that having an opportunity to discuss stigmatizing experiences, fear of stigma, and negative attitudes toward self, helped empower them to manage stigma. This was of particular importance for those who had not disclosed their HIV status to anyone but the health care providers of the infectious disease clinic.

***“I haven’t told anyone about my HIV status. It is so important that the doctor helps me by talking with me about it.” (A 58-year-old woman)***.

## Discussion

The study is a foundation for conceptual models of HIV-specific PROMs in Finland, which is a high-income country with a low HIV prevalence and an aging PLHIV population that has good access to care and treatment. The study identified nine domains for PROMs including understanding viral load, psychological well-being, physical health, social well-being, sexual well-being, uptake of medications, ability to manage other medications with ARVs, stigma and aging with HIV. The study also provides a conceptual framework for the development of PREMs including helping patients understand their health status, providing opportunities for patients to discuss physical health and psychological and sexual well-being, supporting the uptake of ARVs, assisting patients with the use of other types of medications with ARVs, showing care towards patients, and empowering patients against stigma. The study advances the importance of patient-centered care by ensuring the perspectives of PLHIV are at the heart of the model through a co-design methodology [5]. The study provides additional evidence of the importance of approaching the development of HIV PROMs and PREMs from the localized perspective as the set of dimensions of well-being that were identified is a unique mix that cannot be found in any available PROMs and PREMs, which typically include some items but are missing others [4]. The themes identified in this study overlap in many ways as well-being is a complex phenomenon in which different factors influence one another [31]. However, the synergies and overlaps were not presented or analyzed further as the aim of the study was to identify topics that can be developed into PROM and PREM questions. The PROMs and PREMs identified in this study can be considered to be used in similar settings. The findings of this study can be considered in similar low-prevalence settings, with good access to care and treatment and with an aging PLHIV population.

The study did not only show which PROMs should be addressed in clinical care in Finland, but rather how they should be addressed. For example, the concerns about physical health in our study related to chronic conditions and worry about aging. PLHIV may not raise these issues during their consultation in the infectious disease clinic as they may think that these conditions require other specialists. Accordingly, doctors and nurses may need to prompt these topics during consultations. Sexual well-being among PLHIV in our study was also related to a number of concepts such as relationship issues, gynecological well-being as well as reproductive issues that highlight the need for referrals to specialists but also to provide PLHIV with accurate information so that they can make informed choices regarding their sexual well-being.

Our study also indicates that perceived stigma and experiences of stigma influence the well-being of PLHIV in Finland. Stigma is well documented in the global HIV literature [32–38] but is not covered in generic quality of healthcare indicators in Finland, highlighting the importance of the global trend of moving from generic PROMs towards disease-specific PROMS that cover issues of more relevance to specific patient groups [39]. Stigma and discrimination are known to be associated with poor physical and mental health outcomes [40–42], low social support, and reduced income for PLHIV [4035], which makes stigma a crucial issue to address. Our study indicates that PLHIV’s fear of stigma requires empowering them to disclose their HIV status to others if they wish to do so. The study also points out that PLHIV experience stigma and discrimination in their everyday life, which requires powering them to manage stigma. Stigma-related PROM is also a valuable tool for health authorities to monitor stigma in the healthcare setting. In low-prevalence settings, healthcare providers often have little exposure to PLHIV which can increase stigma [43]. Health authorities can develop stigma reduction interventions to target both healthcare providers and PLHIV, such as training of healthcare providers to identify self-stigma and perceived stigma as well as empower patients against them [38, 39, 44, 45].

The study also highlighted the critical role of doctors and nurses to manage the multi-dimensional needs and priorities of PLHIV. They need to have knowledge of various areas and be capable of providing the right support regardless of whether the issues are physical, psychological, health, social, or sexual in nature as trusted sources of information. This is aligned with previous studies that show the importance of doctors and nurses as sources of quality information [32, 33]. Healthcare personnel should be trained to manage the multi-dimensional needs of PLHIV including empathetic approaches to sensitive issues [34]. In addition, healthcare personnel must be sensitized to value all dimensions of well-being instead of focusing efforts solely on viral suppression [35].

The development of the Positive Outcomes HIV PROM, a brief, comprehensive tool for use within routine HIV care, represents a significant step towards a personal outcome approach. The study confirmed the importance of addressing a multidimensional set of factors to capture the well-being of PLHIV and the diverse needs that PLHIV has in the clinical setting [1, 3, 22] (Kall et al. 2020; Hoang 2009; Bristowe et al. 2020). For example, psychological well-being reflects social and sexual well-being, physical health, uptake of medications, and stigma that need to be addressed when developing strategies to support the well-being of PLHIV. Another multi-dimensional factor that came up in the study was the stigma against PLHIV, which is well documented in the global HIV literature [34–36]. It is not covered in generic quality of healthcare indicators in Finland highlighting the importance of the global trend of moving from generic PROMs towards disease-specific PROMS that cover issues of more relevance to specific patient groups [37]. Stigma and discrimination are known to be associated with poor physical and mental health outcomes [38–40]low social support, and reduced income for HIV-positive people [40], which makes stigma a crucial issue to be addressed. Our study showed that stigma was also prevalent in the healthcare setting that requires the sensitization of healthcare personnel against HIV-related stigma in Finland. Previous studies have documented positive outcomes with stigma reduction interventions among healthcare workers that have used known opinion leaders as role models [41]. In addition, healthcare personnel need to be first trained to identify self-stigma and perceived stigma as well as empower patients against them [42].

Measuring the quality and performance of healthcare is a major challenge to improving the efficiency of a health system. This is the first attempt in Finland to develop PREMs and PROMs that adopt a common standard and metric for HIV quality of care, which in turn will enable health officials to directly compare patients’ views of the current delivery of HIV-related health care in Finland. The study indicates that perceived quality of care was strongly linked to provider-patient communication including opportunities to discuss physical, psychological, social, and sexual issues, to get support for ARV uptake, and to feel empowered against stigma. Previous studies indicate that the way that providers communicate with their patients influences the perceived quality of care including transferring information, establishing roles, conveying or reacting to emotions, and balancing power [46, 47]. Improved provider communication is also known to enhance patient-provider relationship-building which may improve patient retention in treatment [48]. Moreover, literature from other parts of the world concludes that patient expectations of healthcare providers are growing [49]. This highlights the importance of ensuring that healthcare providers are capable of meeting these growing expectations. However, this may be a great challenge in the current health systems in Finland where the lack of nursing staff has been increasing gradually [50]. Healthcare providers have also been striving for better salaries and working conditions, most recently during Spring 2022.

The study had limitations. Although study participants were recruited from four different geographic areas, the study did not include participants from the most remote and isolated areas of the country. The data collection tools included some focus group discussions which may not be a suitable setting for everyone to discuss matters of importance to their own life which may have led to some social desirability bias. However, the main data collection tool was one-to-one in-depth interviews during which respondents typically feel more open to discussing sensitive information. Furthermore, there were no representatives of infectious disease clinics during IDIs making it easier also to criticize the health care providers. Further psychometric testing is required to ensure the reliability and responsiveness of the identified concepts.

## Conclusions

This paper described the formative research phase in the development of HIV PROM and PREM questions. It determined the conceptual domains for PROMs and PREMs, which will allow for the development of domain-specific PROM and PREM questions for the national HIV quality of care register. The methodology used for this study allowed people living with HIV to define the issues and domains of importance for them, which highlights advancements in promoting patient-centered HIV care in Finland.

## Electronic supplementary material

Below is the link to the electronic supplementary material.

Supplementary Material 1

## Data Availability

To ensure full anonymity for the participants the transcribed interviews are not possible to share publicly.
