# Supplementary material for: A qualitative study to identify thematic areas for HIV related patient-reported outcome measures (PROM) and patient-reported experience measures (PREM)
Source: J Patient Rep Outcomes. 2023 May 1;7:41. doi: 10.1186/s41687-023-00582-y (PMC10151440; doi:10.1186/s41687-023-00582-y)
Supplement: Supplementary file 1 — Supplementary Material 1 [file 41687_2023_582_MOESM1_ESM.docx]

**Question guide**

1. We would like to start this interview by asking you to list the most important issues which you would also like to share or report to your physician during regular clinic visits.

**Probes:**

- Physical health
- Psychological well being
- Stigma and discrimination
- Side effects of ARVs
- Sexual well being
- Alcohol, drugs, and tobacco use.
- Sleeping problems
- Managing everyday life
- Visiting health services, and HIV experts

1. Any other issues that you can think of that impact your well being?
2. How important do you evaluate the issues you mentioned earlier (ask also about topics that were not mentioned or discussed in (Q1)
3. Can you share some details about the issues you mentioned earlier and explain why they are important to you?

**Probes:** Impact on everyday life

1. Which ones of these issues it is easier to speak with the physicians and which ones are harder?

Probe: Are there differences between what one prefers to discuss with physicians and what ones prefers to discuss with nurses?

1. How do you see as the best way to report these issues to your physician?

**Probe**: A pen and paper form to be filled out before clinic visits? An app that can be filled at home? Face-Face-to-face discussion with physicians.

Thank you for your time
